# High-Resolution Analysis of the Efficiency, Heritability, and Editing Outcomes of CRISPR/Cas9-Induced Modifications of *NCED4* in Lettuce (*Lactuca sativa*)

**DOI:** 10.1534/g3.117.300396

**Published:** 2018-03-29

**Authors:** Lien D. Bertier, Mily Ron, Heqiang Huo, Kent J. Bradford, Anne B. Britt, Richard W. Michelmore

**Affiliations:** * Genome Center; † Department of Plant Biology; ‡ Department of Plant Sciences, Seed Biotechnology Center; § Departments of Plant Sciences, Molecular & Cellular Biology, Medical Microbiology & Immunology, University of California, Davis, CA 95616

**Keywords:** CRISPR/Cas9, NHEJ, targeted mutagenesis, lettuce, germination, thermotolerance, amplicon sequencing

## Abstract

CRISPR/Cas9 is a transformative tool for making targeted genetic alterations. In plants, high mutation efficiencies have been reported in primary transformants. However, many of the mutations analyzed were somatic and therefore not heritable. To provide more insights into the efficiency of creating stable homozygous mutants using CRISPR/Cas9, we targeted *LsNCED4* (*9-cis-EPOXYCAROTENOID DIOXYGENASE4)*, a gene conditioning thermoinhibition of seed germination in lettuce. Three constructs, each capable of expressing Cas9 and a single gRNA targeting different sites in *LsNCED4*, were stably transformed into lettuce (Lactuca sativa) cvs. Salinas and Cobham Green. Analysis of 47 primary transformants (T_1_) and 368 T_2_ plants by deep amplicon sequencing revealed that 57% of T_1_ plants contained events at the target site: 28% of plants had germline mutations in one allele indicative of an early editing event (mono-allelic), 8% of plants had germline mutations in both alleles indicative of two early editing events (bi-allelic), and the remaining 21% of plants had multiple low frequency mutations indicative of late events (chimeric plants). Editing efficiency was similar in both genotypes, while the different gRNAs varied in efficiency. Amplicon sequencing of 20 T_1_ and more than 100 T_2_ plants for each of the three gRNAs showed that repair outcomes were not random, but reproducible and characteristic for each gRNA. Knockouts of *NCED4* resulted in large increases in the maximum temperature for seed germination, with seeds of both cultivars capable of germinating >70% at 37°. Knockouts of *NCED4* provide a whole-plant selectable phenotype that has minimal pleiotropic consequences. Targeting *NCED4* in a co-editing strategy could therefore be used to enrich for germline-edited events simply by germinating seeds at high temperature.

Site-specific or targeted mutagenesis is a powerful method for making specific, intentional changes to the DNA sequence in order to study and alter gene function. Targeted modification of DNA sequences can be achieved by exploiting DNA double-strand break (DSB) repair pathways. DNA repair follows two major pathways: (i) error-prone non-homologous end joining (alt-NHEJ or microhomology-mediated end joining (MMEJ) can introduce insertions or deletions, often leading to frameshift mutations; and (ii) homology-directed repair (HDR) that involves a DNA repair template with complementarity to the target locus to effect a specific modification (Voytas 2013).

CRISPR/CAS-mediated genome editing is revolutionizing foundational and translational research fields, including plant breeding (Podevin *et al.* 2013; Bortesi and Fischer 2014; Puchta 2017). Breeding pipelines include an initial pre-breeding phase to identify genes determining traits of interest and a second breeding phase to introgress these genes into commercial cultivars. Breeding programs currently rely on classical genetic approaches that are laborious and time-consuming for both phases. The time from the phenotypic identification of a gene of interest to release in an improved commercial cultivar is often more than 10 years (Acquaah 2012). Genome editing has multiple applications as an advanced breeding tool. It can be used: (i) to validate the functions of candidate genes using single gRNAs or pooled CRISPR libraries (knockouts); (ii) to modify alleles into more desirable ones (allele editing); (iii) to add or replace genes into cultivars without linkage drag (gene replacement); and (iv) to stack genes at single chromosomal locations so that they will be inherited as a single Mendelian unit (gene stacking) (Kumar *et al.* 2016; Zhao *et al.* 2016). Knockouts are increasingly straightforward in plant species, while allele editing, gene replacements, and stacking need further development to become routine.

The efficiency of obtaining mutations with CRISPR/Cas9 delivered to plants by *Agrobacterium tumefaciens* can be high (Gao *et al.* 2015; Jacobs *et al.* 2015; Zhu *et al.* 2016; Tsutsui and Higashiyama 2017), but many of these mutations are somatic, which makes selection of the desired mutants inefficient (Ma *et al.* 2016). Editing of several plants, including *Arabidopsis*, rice, soybean, and lettuce, has been achieved using Cas9 and Cpf1 ribonucleoproteins (RNPs) transfected into protoplasts (Woo *et al.* 2015; Malnoy *et al.* 2016; Kim *et al.* 2017). Mature plants have been regenerated from Cas9 RNP-edited protoplasts of lettuce (Woo *et al.* 2015). The use of RNPs for editing has advantages, such as reduced off-target activity (Kim 2014) and lack of integration of foreign DNA, which could be beneficial for the commercialization of edited crops. However, there are also disadvantages, as regeneration of mature plants from protoplasts is difficult for some genotypes and species. Also, tissue culture, especially protoplast culture, often results in a high frequency of somaclonal variation (Engler DE 1984; Mou 2011).

Lettuce (*Lactuca sativa*) is a representative diploid member of the Compositae, one of the most successful families of flowering plants (Kesseli and Michelmore 1997; Funk 2005). It is a major crop with a worldwide production of twenty million tons and is a $2.4 billion industry in the United States, mainly in California and Arizona (“Anonymous. Crop Values: 2014 Summary. USDA, http://usda.mannlib.cornell.edu/MannUsda/homepage.do (2015). 11.”). The reference genome sequence of lettuce cv. Salinas and detailed genetic maps are available (Truco *et al.* 2007, 2013; Reyes-Chin-Wo *et al.* 2017). Production of lettuce could benefit from genome editing of numerous traits (Michelmore *et al.* 2017). The *9-cis-EPOXYCAROTENOID DIOXYGENASE4* (*NCED4*) gene is a key regulatory enzyme in biosynthesis of abscisic acid (ABA) (Huo *et al.* 2013). Huo *et al.* (Huo *et al.* 2013) showed that silencing of *LsNCED4* in *L. sativa* cv. Salinas using RNAi prevented inhibition of germination at high temperature but did not have negative pleiotropic effects on normal growth or stress tolerance. Therefore mutants of *LsNCED4* could be commercially valuable in production areas with high temperatures during crop establishment such as in the Imperial and Yuma valleys of southern California and Arizona. In addition, *LsNCED4* could provide a simple, whole-plant selectable marker for use in a CRISPR co-editing strategy; simultaneous targeting of *LsNCED4* and an unrelated gene of interest would enable the selection of plants enriched for editing events in the gene of interest simply by germinating seeds at high temperature.

In order to better understand the timing and nature of editing events in lettuce, we conducted a quantitative study of the efficiency, outcomes, and inheritance of CRISPR/Cas9-induced mutations in the NCED4 gene. We evaluated the timing of modifications induced by CRISPR/Cas9 over multiple generations of stable lettuce transgenics. This revealed frequent mono- and bi-allelic somatic and germline mutations that occurred both in cell culture and later in regenerated plants as well as variation in efficiencies and mutation events induced by different guide RNAs (gRNAs).

## Materials and Methods

### Construct design and transformation

Three gRNAs (gRNA_1, gRNA_2 and gRNA_3; 20 nt-NGG, Table S2 and Figure S1 in File S1) were manually selected in the first half of the intronless *LsNCED4* gene and checked for lack of off-target specificity by BLAST to the lettuce cv. Salinas reference genome (Reyes-Chin-Wo *et al.* 2017). The Gateway-compatible vectors pEn_Chimera and pDe_Cas9 (Fauser *et al.* 2014) were used to generate single gRNA expression vectors (Figure S6 in File S1). The *Cas9* gene is codon optimized for expression in *A. thaliana* and expressed from the parsley ubiquitin (PcUbi) promoter and terminated by the pea 3A terminator (Fauser *et al.* 2014). The gRNA is transcribed by the Pol III AtU6-26 promoter.

pDe_Cas9 was adapted for selection of kanamycin resistance in plants by replacing the *bar* gene cassette with the pNOS-nptII-tNOS cassette. Synthetic gRNA oligos with *Bbs*I overhangs were cloned into *Bbs*I digested pEn_Chimera and the customized gRNA pEn_Chimeras were then transferred into the binary vector pDe_Cas9 by single Gateway LR reactions (Figure S6 in File S1).

The resulting binary vectors were electroporated into *A. tumefaciens* strain LBA4404. Transformations of *L. sativa* cv. Salinas and cv. Cobham Green were done by cocultivating the constructs in *A. tumefaciens* with excised lettuce cotyledons, after which plants were regenerated via somatic organogenesis (Michelmore *et al.* 1987) by the UC Davis Plant Transformation Facility (http://ucdptf.ucdavis.edu/).

Multiple false-positive transgenic T_1_ plants that were resistant to kanamycin but did not contain the complete Cas9 gene occurred in this study (12 out of 59 T_1_ plants). This is likely due to the position of the *NptII* cassette in pDe_Cas9 immediately adjacent to the right T-DNA border. Since T-DNA integration occurs from right to left border, plants with partial T-DNA integrations (Gelvin 2010, 2012) would contain a functional *NptII* cassette, but an incomplete and thus non-functional *Cas9* cassette. The original study with pDe_Cas9 analyzed T-DNA segregation of T_2_ *Arabidopsis* plants by germination in the presence of antibiotic in media (Fauser *et al.* 2014) and so partial T-DNA integrations would have gone unnoticed. The higher number of false positives detected for cv. Cobham Green (11/33) compared to cv. Salinas (1/27), indicates that the plant genotype could possibly influence partial integration of T-DNAs. We have since modified pDe_Cas9 so that the *NptII* cassette is positioned immediately adjacent to the left T-DNA border; no false positives have been detected in subsequent experiments (L. Bertier, M. Ron, A. Britt, R. Michelmore, unpublished data).

### Analysis of calli

Calli were analyzed for editing events three weeks after cocultivation. The frequencies of CRISPR-induced mutations were analyzed in five calli for each combination of gRNA and cultivar (30 total) using three approaches. Genomic DNA was extracted using the GenElute Plant Genomic DNA Miniprep kit (Sigma-Aldrich, St. Louis, MO) and the complete *NCED4* gene (1,737 bp) was amplified using primers NCED4_Salinas_1F and NCED4_Salinas_1R (Table S2) and digested using unique restriction enzymes (gRNA 1: AgeI; gRNA 2: MfeI; gRNA 3: FseI) that had recognition sites at the predicted Cas9 cut sites (Figure S1 in File S1). An undigested band was indicative of Cas9-induced indels that had mutated or deleted the RE recognition site. The undigested 1737 bp *NCED4* amplicon was also Sanger sequenced for 10 calli using primers NCED4_Salinas_1F and NCED4_Salinas_2R. For the same 10 calli, we also generated short ∼150 bp amplicons specific to each gRNA for amplicon sequencing.

### Amplicon sequencing

Calli, T_1_, and T_2_ plants were genotyped by deep amplicon sequencing of the *NCED4* gene. For T_1_ and T_2_ plants, DNA was extracted from leaf tissue of young plants using a custom DNA extraction protocol using a 5 M guanidine isothiocyanate buffer. For T_1_ plants, two sets of primers were designed to generate amplicons of ∼150 bp. Primer pair NCED4_gRNA1_F/NCED4_gRNA1_R spans gRNA1 and primer pair NCED4_gRNA2+3_F/ NCED4_ gRNA2+3_R spans gRNA2 and gRNA3 (Table S2 and Figure S1 in File S1). For T_2_ plants, amplicon libraries were made with the same primer sets as for T1 plants as well as with a third primer set (LDB_226/LDB_227) that amplified a larger ∼380 bp amplicon spanning all three gRNAs. Results shown for T_2_ plants are obtained with primer pair LDB_226/LDB_227. Amplicon libraries were made following the 16S Metagenomic Sequencing Library preparation protocol as provided by Illumina (https://www.illumina.com/content/dam/illumina-support/documents/documentation/chemistry_documentation/16s/16s-metagenomic-library-prep-guide-15044223-b.pdf) with some modifications. For T_1_ genotyping, amplicon and indexing PCRs were done using Phusion Hi-Fidelity polymerase (New England Biolabs, Ipswich, MA). For T_2_ plants, GoTaq polymerase (Fermentas, Waltham, MA) was used for the amplicon and index PCRs. Dual indexing was done using the Nextera XT system (Illumina, San Diego, CA) using 16 i5 indexes (S502-S522) and 24 i7 indexes (N701-N729) enabling multiplexing of 384 individual libraries. SR 100 bp or PE 150 sequencing was done on an Illumina HS3000 or HS4000 platform. Sequence analysis was done using CrispRVariants (Lindsay *et al.* 2016).

Given the recent concerns of index switching during sequencing of dual index multiplexed samples using Illumina platforms (Sinha *et al.* 2017), the pooled libraries were treated with Exonuclease VII (NEB, Ipswich, MA) to remove all residual single-stranded index primers, after which we performed a BluePippin (Sage Science Inc., Beverly, MA) size selection treatment for removal of all fragments smaller than 100 bp. After sequencing, we analyzed the occurrence of index switching in our datasets by demultiplexing the unused index combinations and mapping the reads to the reference. For the two 384-plex libraries analyzed, we calculated an average rate of index switching (the average read number of the used index combinations mapping to the reference divided by the average read number of the unused index combination mapping to the reference) of 0.25 and 0.47%. While these levels of index switching are low and do not impact our results, index switching should be considered and evaluated by demultiplexing the unused index combinations and mapping to the reference sequence(s). Appropriate thresholds for low frequency variants should be set to take possible index switching into account.

### High temperature germination assay

Germination of wildtype seeds from five different years (2006, 2007, 2011, 2014, 2016) was assayed at 25°, 28°, 32°, 35° and 37° by imbibing 75 seeds (3 replications of 25 seeds) per temperature in Petri dishes containing germination paper (Ahlstrom grade 628, Stults Scientific, IL) and 3 mL milliQ water. Germination frequency was recorded after 72 h. Phenotyping was done on T_2_ families from 44 T_1_ primary transformations. As above, 75 seeds (3 replications of 25 seeds) were imbibed at an inhibitory temperature (Cobham Green: 32° and Salinas: 35°) and germination percentages were recorded at 72 h. After 72 h, non-germinated seeds were separated from germinated seedlings and were transferred to room temperature to allow germination. For lines without germination at high temperature, DNA was extracted from leaf tissue of 8 (Cobham Green) or 16 (Salinas) seedlings and a bulk DNA sample was genotyped by amplicon sequencing. For lines having germination at high temperature, DNA was extracted from a young leaf of 16 individual seedlings that either showed germination at high temperature or not, in ratios representative of the total germination frequency. For example if the germination frequency at high temperature for a T_2_ family was 25%, we extracted DNA from 4 seedlings that germinated at high temperature and from 12 seedlings that did not germinate at high temperature. Individual genotypes for all plants were generated using amplicon sequencing.

### Data availability

Amplicon sequencing data for T_1_ and T_2_ plants is available at the SRA (BioProject number PRJNA429829). Figure S1 in File S1 contains an alignment of the four *Ls*NCED paralogs. Figure S2 in File S1 shows the analysis of editing in calli. Figures S3–S5 in File S1 show Sanger sequencing profiles from the calli that were also sequenced by Illumina. Figure S6 in File S1 shows the T-DNA construct. Figure S7 in File S1 shows the temperature/germination relationship of seed lots of different ages for wildtype Cobham Green and wildtype Salinas. Figure S8 in File S1 shows violin plots of the distribution of the percentage of mutated reads for individual T_2_ plants that had different germination phenotypes. Figures S9–S13 in File S2 show mutation profiles derived from amplicon sequencing of individual T_2_ plants from families with germination at high temperature.

## Results

### Selection of gRNAs and transformation

Protospacer sequences for three different gRNAs in the first half of the intronless *LsNCED4* gene were selected that had restriction enzyme recognition sites at or close to the predicted Cas9 cut site, 3 bp upstream of the PAM. The gRNAs were also checked for homology against the other *NCED* paralogs. The three other *NCED* paralogs have a different PAM at the paralogous gRNA sites so that off-target events in these genes will be precluded. (Figure S1 in File S1). This region of the gene was identical in lettuce cvs. Salinas and Cobham Green. No off-target sites having significant sequence homology to the gRNAs were detected by BLAST searches of the lettuce genome (Reyes-Chin-Wo *et al.* 2017). Each gRNA sequence was cloned into pDe_Cas9 (Fauser *et al.* 2014) that had been modified for selection of kanamycin resistance in transgenics. The three resulting constructs were used in independent cocultivations of *A. tumefaciens* with cotyledon explants of cvs. Salinas and Cobham Green. Kanamycin-resistant calli formed within 3 weeks on all explants and plantlets regenerated via somatic organogenesis from 4 weeks after cocultivation. The plants analyzed in this study were derived from independent explants.

### Editing was only detected at low frequency in young calli

Calli were analyzed for editing events three weeks after cocultivation. The frequencies of CRISPR-induced mutations were analyzed in five calli for each combination of gRNA and cultivar (30 total) using three approaches. Genomic DNA was extracted and the complete *NCED4* gene (1,737 bp) was amplified and digested using unique restriction enzymes that had recognition sites at the predicted Cas9 cut sites (Figure S1 in File S1). An undigested band was indicative of Cas9-induced indels that had mutated or deleted the recognition site. Some calli had a faint undigested band that represented <10% of the total amplicon (Figure S2 in File S1). One callus (Sal_3_c5) showed amplification of an additional band at ∼850 bp, indicating a ∼900 bp deletion (Figure S2Ca-b in File S1). The undigested amplicons from 10 calli with possible indels were Sanger sequenced. The majority of chromatograms showed no clear evidence of multiple sequences, indicating that there had been little or no editing (Figures S3–S5 in File S1). The ∼900 bp deletion in callus Sal_3_c5 was evident starting around 100 bp before the Cas9 cut site for gRNA 3 (Figure S5 in File S1). The restriction enzymes were suboptimal for detecting indels induced by gRNA 1 and 2 because the recognition site did not span both sides of the predicted Cas9 cut site. Therefore, libraries of amplicons were Illumina sequenced for the same 10 calli (Figure S2D-F in File S1). Multiple mutations were detected at low frequency for all 10 calli; the highest frequency was ∼15% of total reads. For gRNA 1, 13 different indels were detected at a threshold of 1% of reads for at least one callus sample, the majority of which were deletions of 1 to 12 bp. For gRNAs 2 and 3, only three alleles were detected, two of which were 1 bp insertions. The higher frequency of deletions for gRNA 1 is likely due to 6 bp of microhomology (CCAACCN_11_CCAACC) at the protospacer region. All three methods of analysis indicated that the initial frequency of editing in calli was low. This could have been due to low Cas9 activity or DNA repair processes in rapidly dividing cells, or due to technical reasons such as differences in promoter activities driving either the *Cas9* gene or the gRNAs relative to the *Nos* promoter expressing the *NPTII* selectable marker gene in calli. Also, at least some of the kanamycin-resistant cells in the calli would have lacked a functional *Cas9* gene due to the location of the *NPTII* gene next to the right T-DNA border (see below and Methods for details).

### High frequency of mono- and bi-allelic T_1_ plants indicates that Cas9 was active in the founder cells

**Figure 1:**
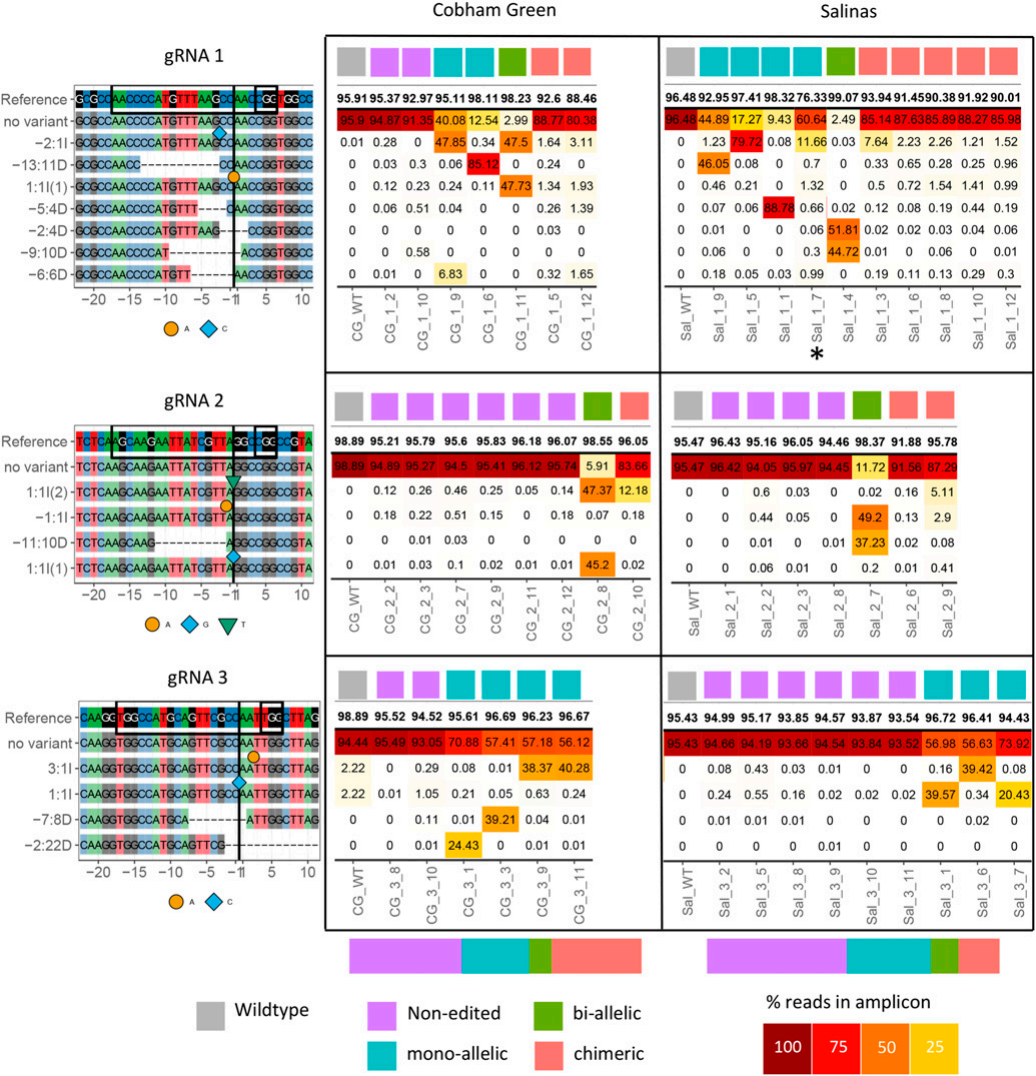
Amplicon sequencing analysis of 47 CAS9 positive T_1_ plants. Each row represents an allele, with the reference allele on top. The 23bp gRNA sequence is circled with a black box, with the PAM circled by the small black box. The predicted CAS9 cut site is indicated with a black line. Each column represents the sequence read distribution in a young leaf of a single T_1_ plant. A wildtype control is shown on the left of each panel. Plants were divided into four genotypic classes according to their mutation profiles. 43% of plants showed no editing (<5% mutated reads in the amplicon, indicated in purple); 28% were mono-allelic with >20% reads for a single allele indicating one early event (indicated in blue); 8% were bi-allelic (2 early events with >20% reads, indicated in green), and 21% were chimeras (containing multiple low frequency alleles adding up to >5% of the total reads, indicated in red). Bar on bottom of plots represents distribution of NCED4 genotypes for all CAS9 positive Cobham Green (left) and Salinas (right) T1 plants. * For plant Sal_1_7, a mono-allelic 44bp deletion was present but only detected in T_2_ plants where a larger amplicon was sequenced (see Figure S11 in File S2). Plant abbreviation is Genotype_gRNA_plant. Genotypes: CG = Cobham Green; Sal = Salinas.

A total of 59 primary transgenic plants (T_1_) were derived for the six gRNA/cultivar combinations (Table S1). Although all plants were resistant to kanamycin, PCR amplification of the *Cas9* gene was negative for 12/59 plants (11/32 cv. Cobham Green; 1/27 cv. Salinas), indicating that these plants contained only partial T-DNA integrations and lacked a full *Cas9* cassette. This was likely due to the position of the *NptII* cassette in pDe_Cas9, immediately adjacent to the right T-DNA border (see Methods for details). A variety of editing frequencies were detected in the 47 *Cas9* positive transformants. Short ∼150 bp fragments containing each of the gRNA target sites were amplified from DNA extracted from an early leaf for all 59 T_1_ plants (Figure 1, Figure S1 in File S1 and Table S1). Libraries from each individual leaf were Illumina sequenced to an average coverage of 5,000 reads. None of the *Cas9*-negative plants contained editing events in amplicons from T_1_ leaves, indicating that T-DNA integration rather than transient T-DNA expression was necessary for detectable mutations. Out of 47 *Cas9*-positive T_1_ plants, 27/47 (57%) contained at least 5% edited reads in the *NCED4* amplicon from leaf tissue; 20/47 (43%) leaves had <5% edited *NCED4* reads. No difference in editing efficiency was detected between the two genotypes (Cobham Green: 11/21; Salinas: 16/26). Mutation efficiency was highest for gRNA 1 with 15/17 *Cas9*-positive plants containing editing events at the target site. Only 5/15 plants were edited for gRNA 2 and 7/15 plants were edited for gRNA 3. All alleles except one (-6:6D for gRNA 1; Figure 1) were frame-shift mutations. The most common mutations were single base-pair insertions (A/T or C/G) and deletions (4 to 22 bp). Seven different mutations above a threshold of 5% reads for at least one T_1_ leaf were detected for gRNA 1, while four different mutations were detected for both gRNA 2 and gRNA 3.

Four categories of mutants could be identified from the mutation patterns of the T_1_ plants (Figure 1) and segregation patterns in T_2_ families (Figures S9–S13 in File S2). Out of 47 T_1_ plants, 4 plants (8%) were bi-allelic mutants, 13 (28%) were mono-allelic mutants, 10 (21%) were chimeras that contained multiple alleles at low frequency in the T_1_ leaf and 20 (43%) had no editing. None of the chimeric T_1_ plants contained more than three alleles (at a threshold of 5% reads per allele), suggesting that somatic Cas9 activity was low. However, in contrast to the T_1_ plants, leaves of T_2_ progeny often had more than three alleles, indicating high somatic Cas9 activity in the T_2_ generation (Figures S9–S13 in File S2).

### Knockout of NCED4 resulted in high temperature germination and can serve as a whole-plant selectable marker for germline editing events

The germination performance of wild type seed was established for each genotype over a range of temperatures between 25 and 37° (Figure S7 in File S1). Germination of younger seed lots (≤5 years old) of cv. Salinas was unaffected until 32°; at 35° there was a sharp decline in germination percentage and almost no germination was observed at 37°. Older seed lots were slightly more sensitive to warm temperatures. Germination of cv. Cobham Green seeds was more sensitive to temperature; germination of more recent seed lots was unaffected at 25°, less than 50% at 28°, and less than 10% at 30° and above for all seed lots.

**Figure 2:**
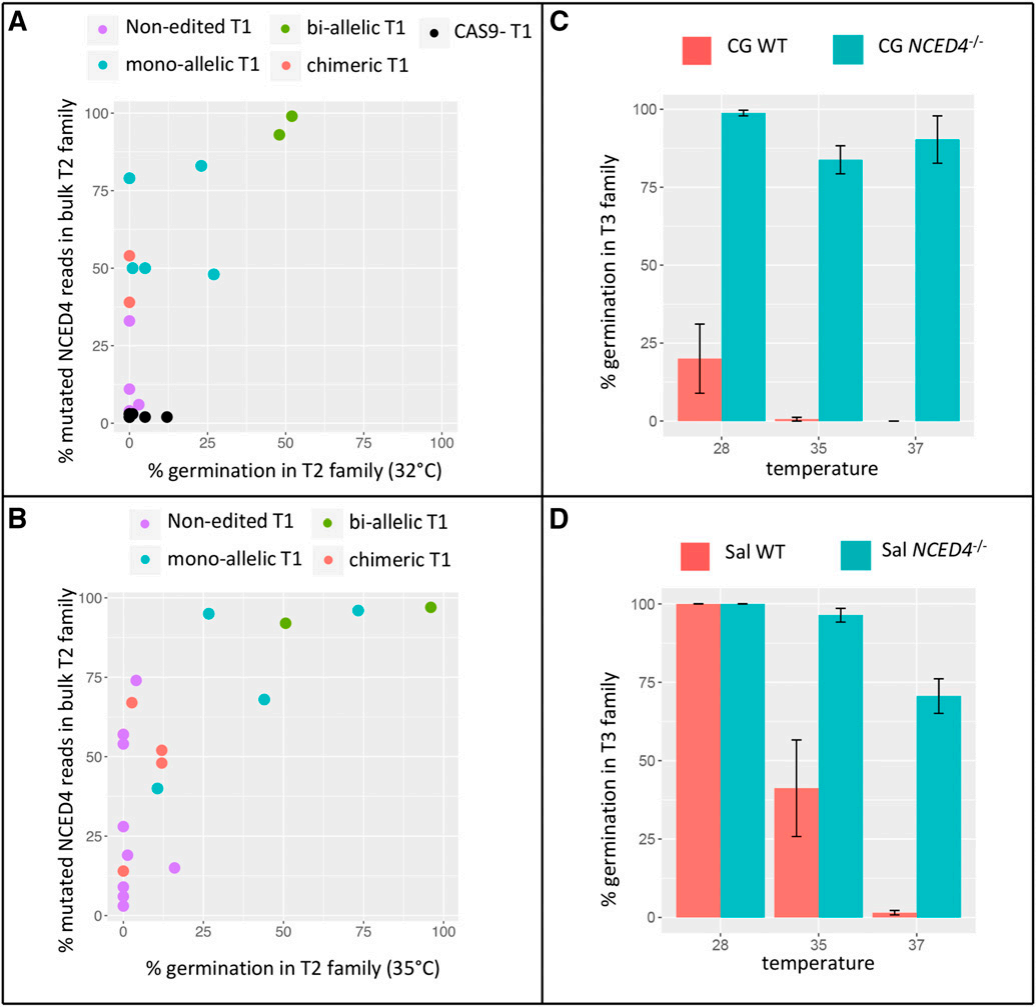
Germination at high temperature for T_2_ and T_3_ families. A, Germination of 23 Cobham Green T_2_ families germinated at 32°C; B, germination of 20 Salinas T_2_ families germinated at 35°C. Germination is plotted against the percentage of edited reads in the bulk T_2_ family. Color coding refers to the T_1_ leaf genotype. C, D: Germination of T_3_ families at different temperatures for Cobham Green (C, mean of 6 T_3_ lines) and Salinas (D, mean of 6 T_3_ lines) compared to wildtype (mean of 3 lines from different years for each cultivar).

To assay the germination thermosensitivity of potentially edited lines, all 59 T_1_ plants were selfed and the T_2_ seeds harvested. Almost a third of T_1_ plants (16/59) were partially or completely sterile (≤150 seeds/plant); sterility is common in lettuce plants derived from tissue culture and was not correlated with the *NCED4* genotype in the leaves of *Cas9*-positive T_1_ mother plants and three of these plants did not have a functional *Cas9* gene (Table S1). Twenty T_2_ seeds of the 43 non-sterile T_1_ plants were pre-screened for viability by germination at room temperature. After 96 h, viability was >99%. Seed thermoinhibition at high temperature was further evaluated by germinating T_2_ families of cv. Cobham Green at 32° and of cv. Salinas at 35°. T_2_ families of the same 43 T_1_ plants (23 of cv. Cobham Green and 20 of cv. Salinas) were evaluated for germination at high temperature by imbibing 75 seeds per T_2_ family at 32° for cv. Cobham Green and at 35° for cv. Salinas. Germination data were collected at 72 h (Table S1 and Figure 2) and germinated seedlings were separated from non-germinated seed. The latter were shifted to room temperature and allowed to complete germination. Genotypes were then determined for individual T_2_ seedlings in families where germination was greater than zero at high temperature. For T_2_ families with no germination at high temperature, DNA was extracted from young leaves, pooled, and amplified; a library representing equal amounts of DNA from each plant was then sequenced.

**Figure 3:**
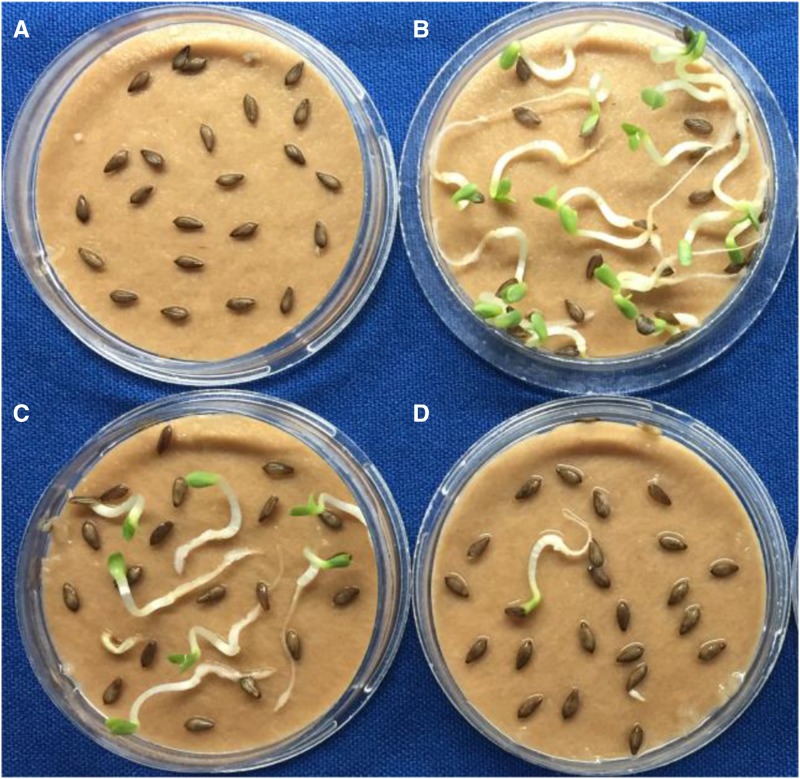
High temperature germination phenotype. Germination of 4 Salinas T2 families after 72 h at 35°C. A: Sal_2_2, non-edited T1; B: Sal_2_7, bi-allelic T1; C: Sal_1_9, mono-allelic T1; D: Sal_3_6, mono-allelic T1.

T_2_ progeny derived from T_1_ plants with different mutant profiles in their leaves exhibited a range of high temperature germination frequencies as illustrated in Figure 2 and Figure 3.

The consequence of editing *NCED4* was evaluated by plotting the germination frequency against total editing in each T_2_ family (percentage of edited reads in amplicons bulked prior to sequencing or *in silico*; Figure 2A-B). For both cultivars, the highest germination frequency was detected for T_2_ families with mono- and bi-allelic T_1_ leaf genotypes, consistent with an inverse correlation between *NCED4* expression and germination at high temperature. Only one bi-allelic line germinated close to 100%. Three out of four T_2_ progeny from plants with bi-allelic T_1_ leaf genotypes had germination percentages of around 50%; genotypes of these individual T_2_ plants revealed a 1:2:1 segregation (homozygous for allele 1: bi-allelic: homozygous for allele 2) indicating that the high temperature germination phenotype was not absolute and not all homozygous mutants germinated at high temperature (Figure S8 in File S1). Germination of the T_2_ progeny with mono-allelic T_1_ leaves varied between 0% and 73% (average 21%, n = 8). Germination of T_2_ families with chimeric T_1_ leaves ranged between 0% and 12% (average 4%, n = 7). None of the T_2_ lines derived from a chimeric T_1_ had a high germination percentage, indicating that T_1_ plants with chimeric profiles contained mostly somatic mutations that were not inherited through the germline. Germination of T_2_ progeny with non-edited T_1_ leaves was low (average 2%, n = 14). For these T_2_ families derived from non-edited T_1_ plants (but with *Cas9*), substantial amounts of editing was observed in T_2_ leaves (average 23%, n = 14), indicating that *Cas9* remains active and new mutations can arise in the *NCED4* gene in T_2_ plants.

T_3_ progeny from T_2_ plants that were homozygous for *NCED4* knockouts consistently showed a complete loss of thermoinhibition of germination regardless of whether the T_2_ progenitor had germinated at high temperature in the previous generation, indicating that non-genetic factors influenced germination of T_2_ individuals. T_3_ seed was collected from 11 individual T_2_ plants that were homozygous for the *NCED4* gene and either positive or negative for the *Cas9* gene. Germination tests were performed at 28, 35, and 37°. All homozygous *NCED4* mutants in both genotypes showed significantly higher germination percentages at 35 and at 37° Compared to wild type (Figure 2B-C). At 37°, mutant lines in both genotypes had average germination percentages above 70%, while germination was completely inhibited in wild type plants.

### Mutational events are not random but distinctive for each gRNA

**Figure 4:**
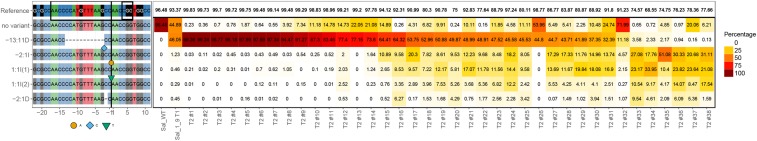
Mutation profiles of 38 T_2_ progeny of Sal_1_9. Each row represents an allele, with the reference allele on top. The 23bp gRNA sequence is circled with a black box, with the PAM circled by the small black box. The predicted *Cas9* cut site is indicated with a black line. Each column represents the sequence read distribution in a young leaf of a single T_2_ plant. A wildtype control is shown on the left, followed by the mutation profile of Sal_1_9, the T_1_ mother plant. Plant abbreviation is Genotype_gRNA_plant. Genotypes: CG = Cobham Green; Sal = Salinas.

Genotyping of individual T_2_ plants revealed that the types of mutations were not random, but specific to the gRNA (Figures S9–S13 in File S2). Since a mutant allele inherited through the germline should be represented by either 50% (heterozygous) or 100% (homozygous) of the reads in the amplicon, any low frequency alleles (<50%) in leaves of T_2_ plants represent new, somatic events due to continued *Cas9* expression. This is illustrated by the mutation profiles of 38 T_2_ progeny of line Sal_1_9 (Figure 4). The T_1_ plant containing gRNA1 had a single 11 bp deletion in the young leaf that represented 46% of the reads in the amplicon. Segregation in the T_2_ progeny indicated that this was a mono-allelic germline mutation (9/38 were homozygous wild types, 9/38 were homozygous mutants and 20/38 were heterozygotes). The *Cas9* gene was still present in all but two of the 38 T_2_ plants, one of which was a homozygous mutant. Therefore new alleles could have arisen in 28/38 plants. In all of these 28 T_2_ plants, the same events (a 1 bp C and a 1 bp A insertion) that were detected most frequently in the 17 other T_1_ plants for gRNA 1, occurred independently. Similarly, for 16 T_2_ progeny of T_1_ plant Sal_3_8 containing gRNA 3, which had no detectable mutations in the T_1_ leaf (Figure S13 in File S2), the same mutations (a 1 bp C, A or T insertion) arose independently in multiple T_2_ individuals. Moreover, for gRNA 3, mutation patterns seem to be especially reproducible in T_2_ sibs (Figures S12 and S13 in File S2). This repeatability indicates that the outcomes of NHEJ repair are not random but influenced by the gRNA sequence.

## Discussion

Multiple strategies for inducing CRISPR/Cas9-mediated mutations in plants have been reported (Schiml and Puchta 2016; Puchta 2017). Most commonly, Cas9 and one or more gRNAs have been combined in a single expression construct, which was delivered by *A. tumefaciens* into embryogenic callus or somatic explants. Different parameters can affect the mutation efficiency, including Cas9 codon optimization (Ma *et al.* 2016), promoters and terminators controlling expression of Cas9 and gRNA (Bortesi and Fischer 2014), transformation procedure (Altpeter *et al.* 2016), callus culture time (Mikami *et al.* 2015), gRNA protospacer sequence (Doench *et al.* 2016; Haeussler *et al.* 2016), and possibly other factors.

We obtained efficient germline editing in lettuce using Cas9 optimized for expression in *A. thaliana* expressed from the parsley ubiquitin (PcUbi) promoter and a single gRNA driven by the pol III AtU6-26 promoter that was mediated by cocultivation of *A. tumefaciens* with cotyledon explants. Although low frequencies of editing were detected in callus samples analyzed three weeks post *Agrobacterium* cocultivation, amplicon sequencing of young T_1_ leaves revealed 13 independent mono-allelic and four bi-allelic plants out of 47 Cas9-positive regenerants. At the time of sampling, callus had been under selection for antibiotic resistance for 18 days. The early sampling time could have been a factor in the low frequency of editing because there can still be some non-transgenic callus this early in selection (D. Tricoli, personal communication). The plants are likely exhibiting more editing than the callus because of the continual selection for the presence of the T-DNA over the 4 to 5 month period. Amplicon sequencing of 368 T_2_ plants derived from 23 T_1_ lines provided a high-resolution analysis of mutation profiles and inheritance patterns. Individual genotypes of T_2_ plants derived from the mono- and bi-allelic T_1_ plants showed clear segregation of the mutated alleles, indicating that the mutations detected in the T_1_ leaves had been inherited through the germline. Average germination at high temperature was highest for the T_2_ families derived from bi-allelic and mono-allelic T_1_ plants. None of the 11 T_2_ families derived from chimeric T_1_ plants showed high germination at high temperature. Although average germination in seeds of T_2_ families derived from non-edited T_1_ plants was low, substantial amounts of editing were detected in the young T_2_ leaves, indicating that there had been a burst of Cas9 activity in the early growth stages of the T_2_ seedlings. This could be due to increased promoter activity in young tissue or to the high temperature germination treatment itself because Cas9 was recently reported to work more efficiently in plants during heat stress (Le Blanc *et al.* 2017). In combination, these results indicate that Cas9 activity and NHEJ was strong during early regeneration of explants or early development of seedlings but weaker in callus and in established leaf tissue.

Our data also provide insights into mutational outcomes of NHEJ in lettuce. The most common mutations were 1 bp insertions that could be any base pair. In addition to insertions, deletions were detected for all three gRNAs, but were more prevalent with gRNA 1, which is likely due to 6 bp of microhomology (CCAACCN_11_CCAACC) in the protospacer region. When microhomologies are present in the vicinity of the DSB, MMEJ is often the dominant mechanism for DSB repair (Rodgers and Mcvey 2016). Repair outcomes were not random, but rather were consistent for each gRNA. Mutation signatures from sustained somatic Cas9 activity seemed to be particularly reproducible in T_2_ sibs. In mammalian cell lines, repair outcomes after CRISPR/Cas9-induced double strand breaks were also not random, but dependent on the gRNA, and independent of genomic location and cell line (Van Overbeek *et al.* 2016). Similarly, independent hairy roots and somatic embryos of soybean had consistent mutation signatures with the same gRNA. These data are indicative of an unknown, controlled mechanism governing the types of mutations that are favored at a given target (Jacobs *et al.* 2015).

Off-target activity was not assessed in this study; however, care was taken to avoid off-target activity in the three other *NCED* paralogs by selecting gRNAs that differed in the PAM regions in the parologous genes. Off-target activity elsewhere in the genome is a possibility, although no off-target matches were found that had complete homology in the seed region (9bp upstream of the PAM). Somaclonal variation in any plant that has gone through tissue culture will likely be higher than off-target effects. Backcrossing would remove both somaclonal variation and any off-target effects.

Our phenotypic and genotypic data indicate that the *NCED4* gene can be used as a selectable marker for germline editing events. Although efficiency of mutagenesis has been shown to be high in multiple species, many mutations in the T_1_ plant are somatic, making selection of the desired inheritable mutants a burdensome process. The most desirable outcome is a bi-allelic germline knockout mutation in the primary transformant. Selfing will then give rise to homozygous or bi-allelic T_2_ mutated progeny with a 3:1 ratio of *Cas9*-positive *vs.* negative segregants. Mono-allelic T_1_ germline mutants will give rise to Cas9-free, homozygous mutants at a ratio of 15:1, assuming no linkage between the T-DNA and the target gene. High frequencies of co-editing have been reported in *C. elegans* (Arribere *et al.* 2014; Kim *et al.* 2014; Mouridi *et al.* 2017). Therefore, the occurrence of germline editing of the *NCED4* gene is expected to be correlated with germline edits of an unrelated target gene. Consequently, selecting for edits of the *NCED4* gene by germinating seeds at high temperature may enrich for desired edits in other genes that are more difficult to screen for phenotypically.

Germination thermotolerance due to inactivation of *NCED4* provides a useful whole-plant selectable phenotype that has little if any pleotropic effects on growth or stress tolerance (Huo *et al.* 2013). However, the level of thermotolerance can vary between genotypes and among seed lots of the same genotype. Germination of seeds of wildtype cv. Cobham Green, a butterhead cultivar, was much more thermosensitive than seeds of cv. Salinas, a crisphead cultivar. Therefore, it will be necessary to determine the optimal temperature for selection of editing events in *NCED4* for each genotype. In addition, lines of the same genotype that are grown under different conditions can have different sensitivities to temperature (Sung *et al.* 1998; Kozarewa *et al.* 2006). Screening efficiency may be enhanced by maturing seeds at lower temperatures to maximize high temperature sensitivity in non-edited seeds. Furthermore, fresh seed (germinated immediately after seed maturation) was more sensitive to high temperature than seed that had been stored for a few months (data not shown).

Until recently, cloning of causal genes for agriculturally important traits has been a lower priority for crop improvement than obtaining genetically linked markers. Once a gene had been mapped to a sufficiently small genomic region, closely linked markers have been adequate for marker-assisted selection to introgress the gene into elite genotypes in breeding programs. However, precise introgressions or alterations in existing alleles using genome editing depend upon the availability of cloned causal genes. Our data show that CRISPR/Cas9 is an efficient tool to aid in gene identification in lettuce by creating single-gene heritable knockouts in one generation. *LsNCED4* could also be used in a co-editing strategy aimed to simplify selection of mutations in candidate genes with more complex phenotypes.

In conclusion, we have demonstrated the utility of the CRISPR/Cas9 system for generating gene knock-outs in lettuce. Experiments to identify candidate genes for resistance to downy mildew caused by *Bremia lactucae* and a bacterial root rot caused by *Rhizomonas suberifaciens* using the *NCED4* co-editing strategy are underway. In addition, we are extending the system to achieve allele editing, gene replacements and gene stacking using the insights gained in this study.

## Supplementary Material

Supplemental Material is available online at www.g3journal.org/lookup/suppl/doi:10.1534/g3.117.300396/-/DC1.
