# Supplementary material for: High-Resolution Analysis of the Efficiency, Heritability, and Editing Outcomes of CRISPR/Cas9-Induced Modifications of NCED4 in Lettuce (Lactuca sativa)
Source: G3 (Bethesda). 2018 Mar 29;8(5):1513–21. doi: 10.1534/g3.117.300396 (PMC5940144; doi:10.1534/g3.117.300396)
Supplement: Supplementary file 3 [file 1513FileS1.pptx]

## Slide 1
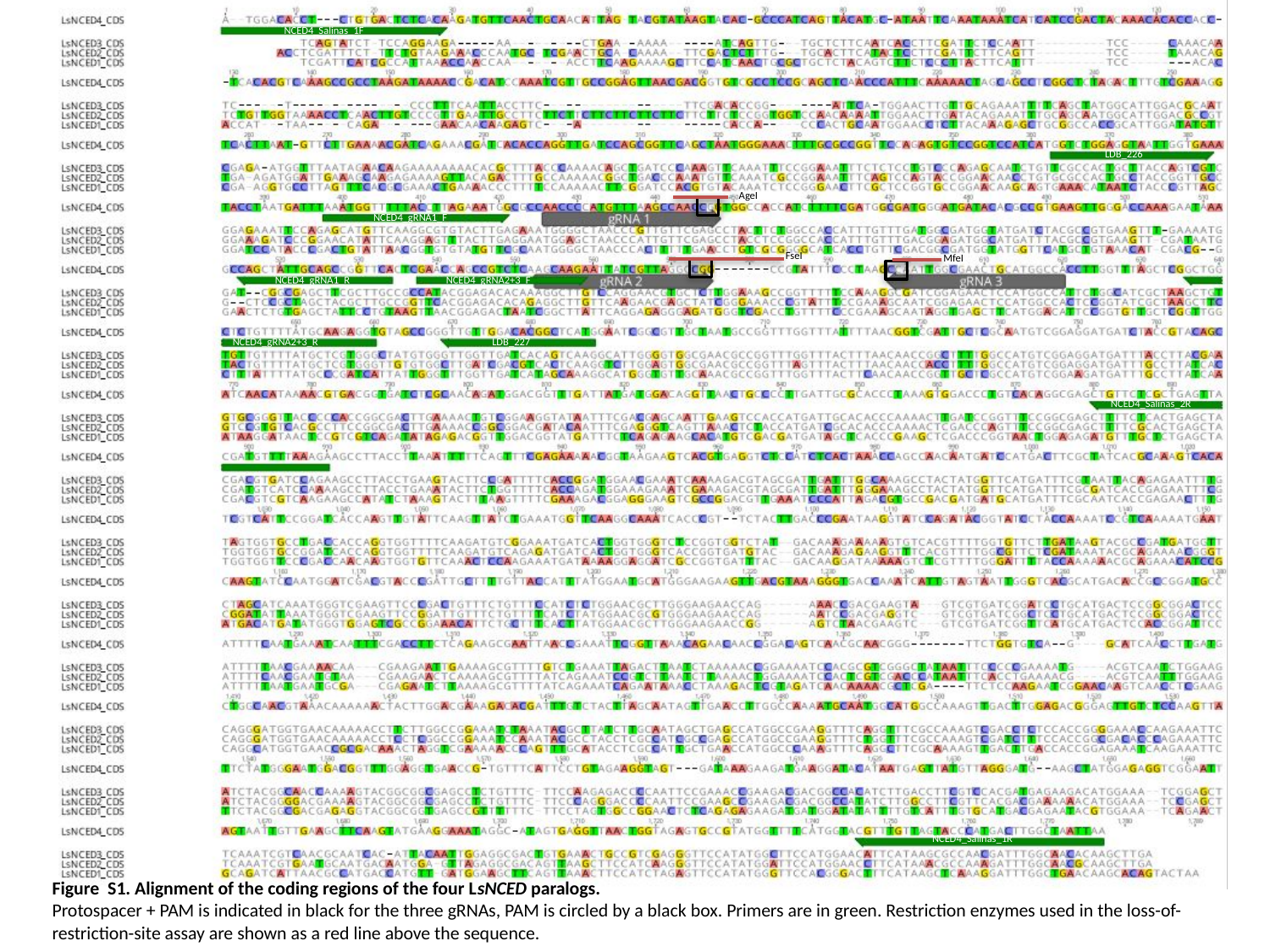

NCED4_Salinas_1F
LDB_226
AgeI
NCED4_gRNA1_F
FseI
MfeI
NCED4_gRNA2+3_F
NCED4_gRNA1_R
NCED4_gRNA2+3_R
LDB_227
NCED4_Salinas_2R
NCED4_Salinas_1R
Figure S1. Alignment of the coding regions of the four LsNCED paralogs.
Protospacer + PAM is indicated in black for the three gRNAs, PAM is circled by a black box. Primers are in green. Restriction enzymes used in the loss-of-restriction-site assay are shown as a red line above the sequence.

## Slide 2
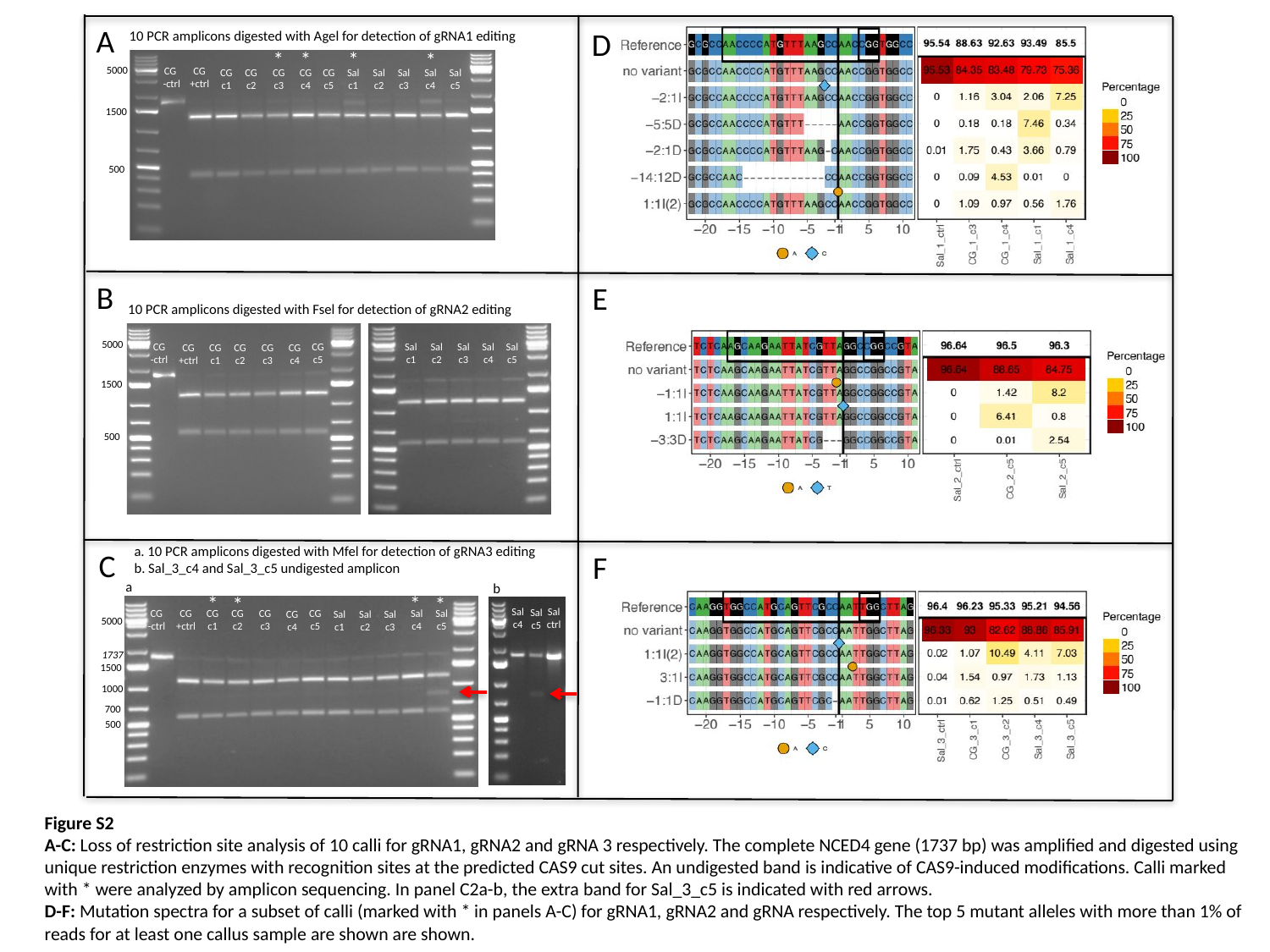

A
10 PCR amplicons digested with Agel for detection of gRNA1 editing
*
*
*
*
CG
c1
CG
c2
CG
c3
CG
c4
CG
c5
Sal
c1
Sal
c2
Sal
c3
Sal
c4
Sal
c5
CG +ctrl
CG
 -ctrl
5000
1500
500
D
B
E
10 PCR amplicons digested with Fsel for detection of gRNA2 editing
Sal
c1
Sal
c2
Sal
c3
Sal
c4
CG
c5
Sal
c5
CG
+ctrl
CG
c1
CG
c2
CG
c3
CG
c4
CG
-ctrl
*
*
5000
1500
500
a. 10 PCR amplicons digested with Mfel for detection of gRNA3 editing
b. Sal_3_c4 and Sal_3_c5 undigested amplicon
C
F
a
b
*
*
*
*
Sal
ctrl
Sal
c4
Sal
c5
Sal
c4
CG
c5
Sal
c5
CG
+ctrl
CG
c1
CG
c2
CG
c3
CG
c4
Sal
c1
Sal
c2
Sal
c3
CG
-ctrl
5000
1500
500
1737
1000
700
Figure S2
A-C: Loss of restriction site analysis of 10 calli for gRNA1, gRNA2 and gRNA 3 respectively. The complete NCED4 gene (1737 bp) was amplified and digested using unique restriction enzymes with recognition sites at the predicted CAS9 cut sites. An undigested band is indicative of CAS9-induced modifications. Calli marked with * were analyzed by amplicon sequencing. In panel C2a-b, the extra band for Sal_3_c5 is indicated with red arrows.
D-F: Mutation spectra for a subset of calli (marked with * in panels A-C) for gRNA1, gRNA2 and gRNA respectively. The top 5 mutant alleles with more than 1% of reads for at least one callus sample are shown are shown.

## Slide 3
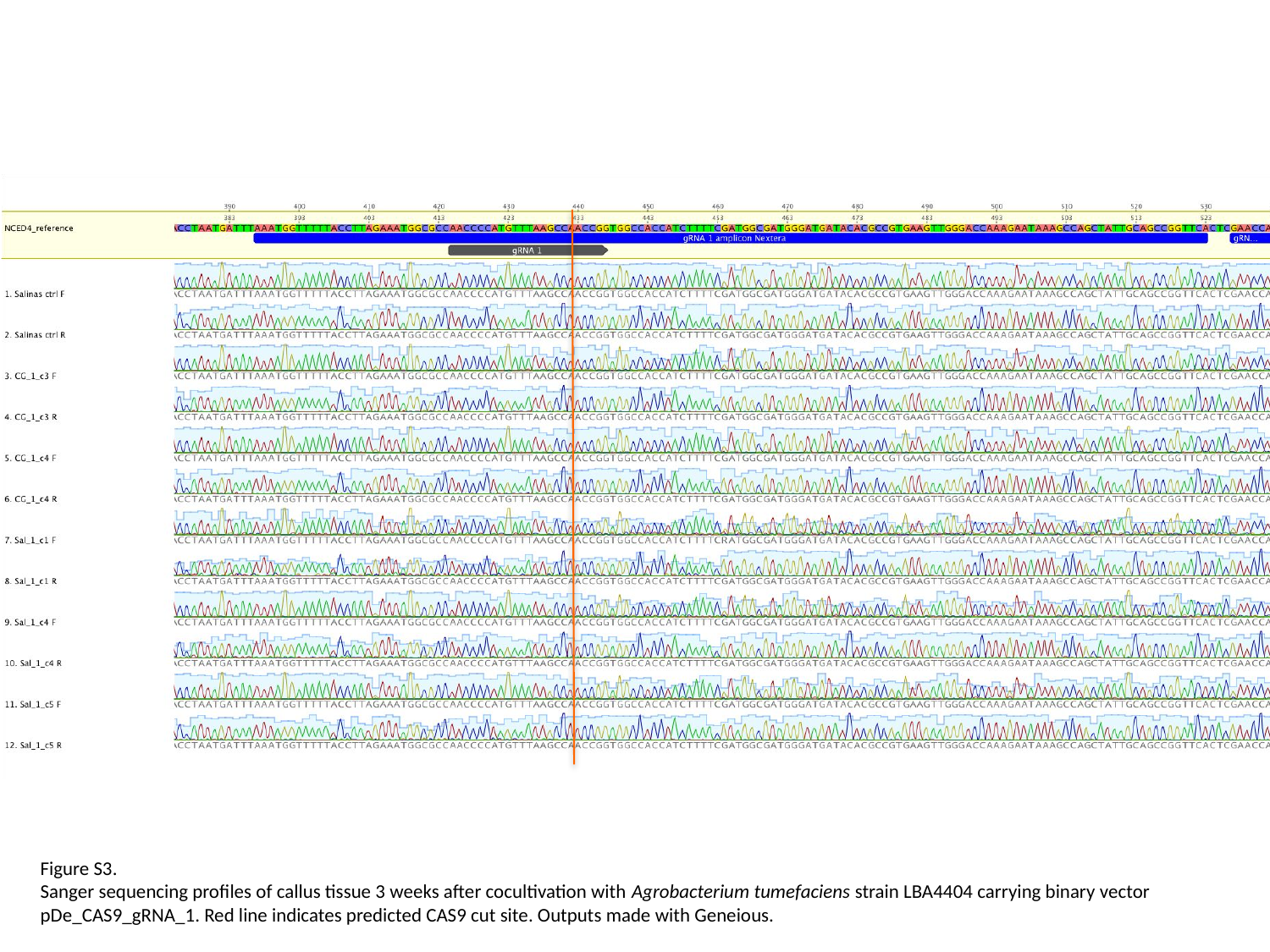

Figure S3.
Sanger sequencing profiles of callus tissue 3 weeks after cocultivation with Agrobacterium tumefaciens strain LBA4404 carrying binary vector pDe_CAS9_gRNA_1. Red line indicates predicted CAS9 cut site. Outputs made with Geneious.

## Slide 4
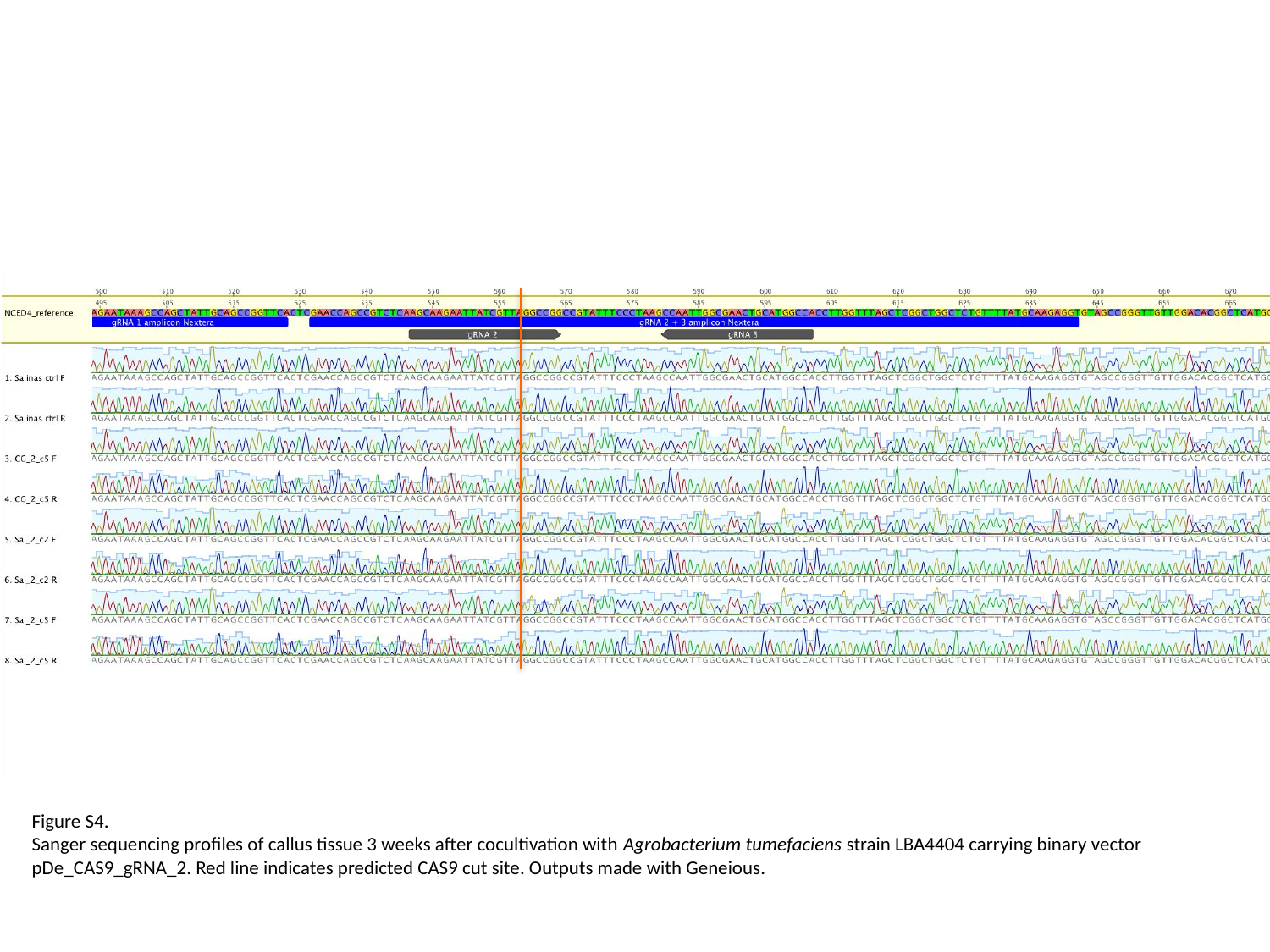

Figure S4.
Sanger sequencing profiles of callus tissue 3 weeks after cocultivation with Agrobacterium tumefaciens strain LBA4404 carrying binary vector pDe_CAS9_gRNA_2. Red line indicates predicted CAS9 cut site. Outputs made with Geneious.

## Slide 5
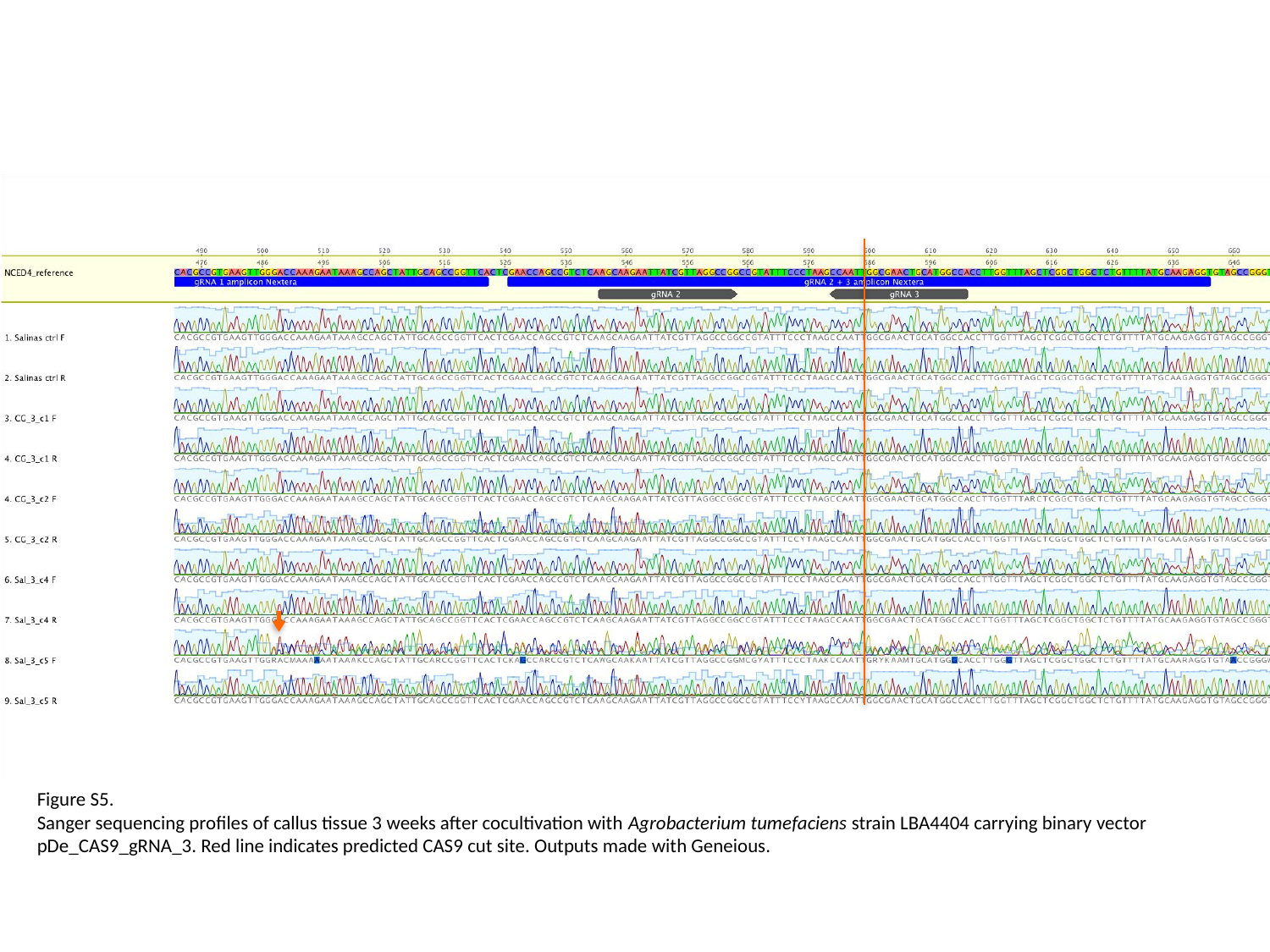

Figure S5.
Sanger sequencing profiles of callus tissue 3 weeks after cocultivation with Agrobacterium tumefaciens strain LBA4404 carrying binary vector pDe_CAS9_gRNA_3. Red line indicates predicted CAS9 cut site. Outputs made with Geneious.

## Slide 6
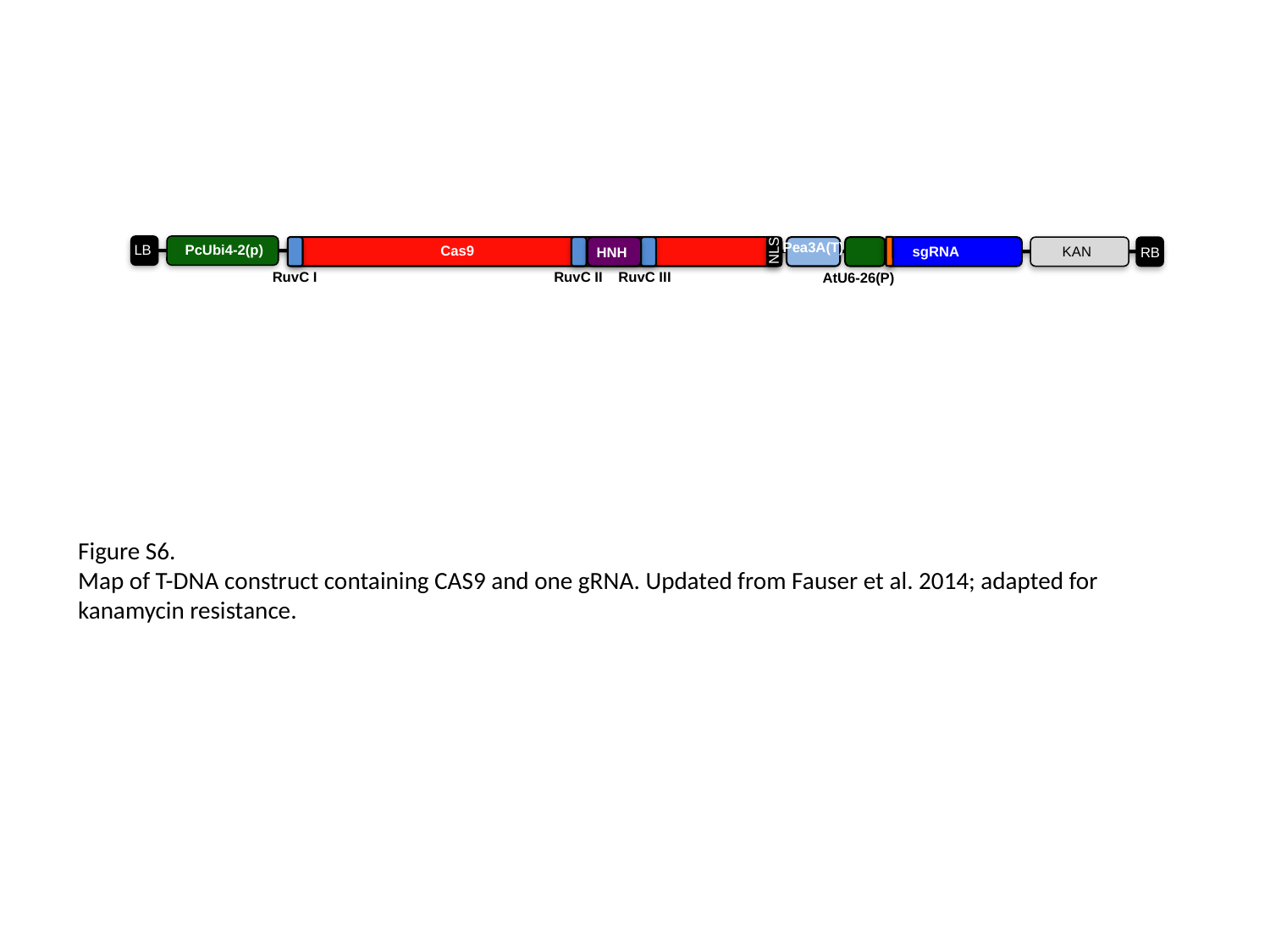

Pea3A(T)
PcUbi4-2(p)
LB
Cas9
KAN
sgRNA
AtU6-26(P)
HNH
RB
NLS
RuvC II
RuvC I
RuvC III
Figure S6.
Map of T-DNA construct containing CAS9 and one gRNA. Updated from Fauser et al. 2014; adapted for kanamycin resistance.

## Slide 7
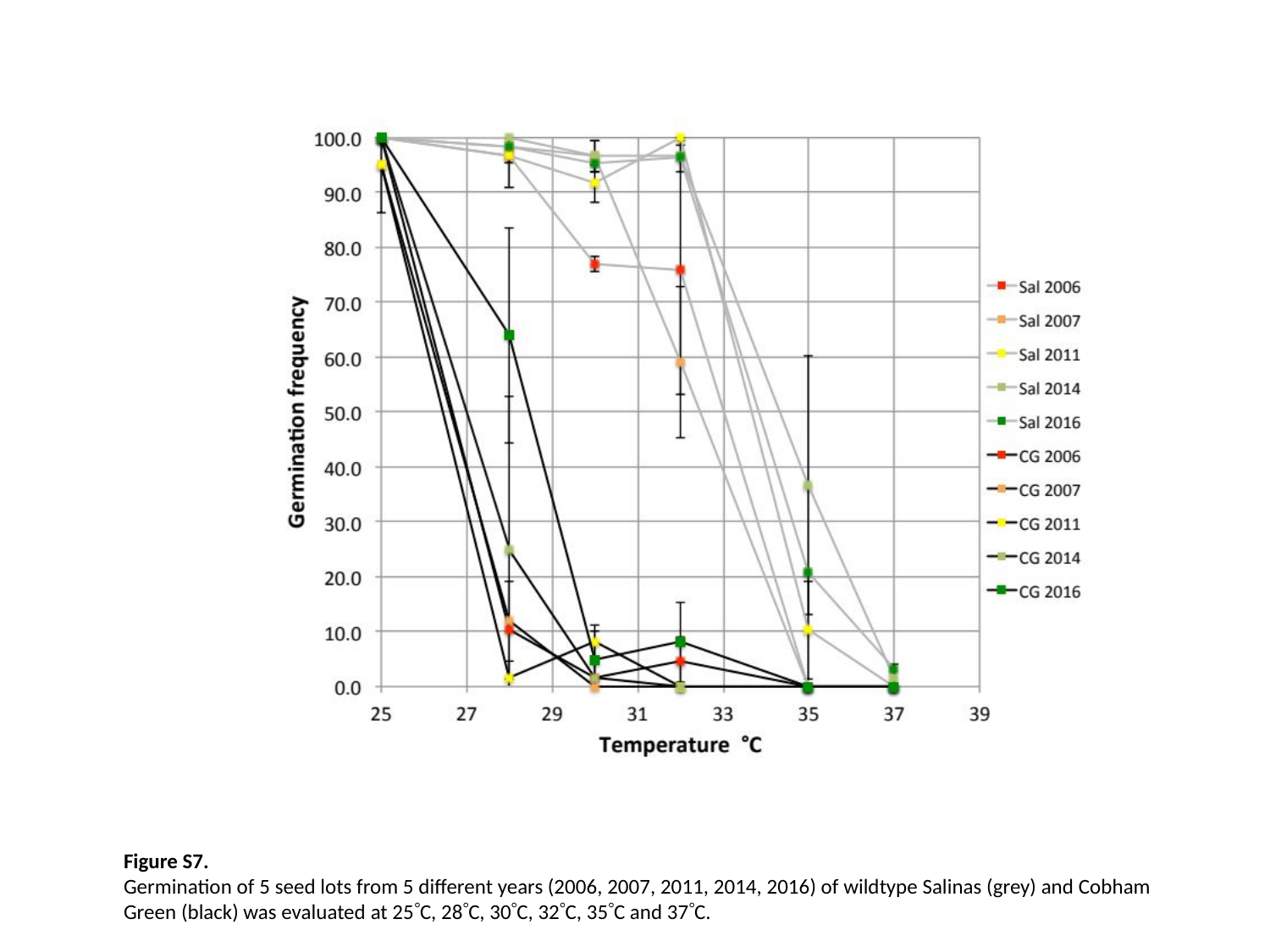

Figure S7.
Germination of 5 seed lots from 5 different years (2006, 2007, 2011, 2014, 2016) of wildtype Salinas (grey) and Cobham Green (black) was evaluated at 25°C, 28°C, 30°C, 32°C, 35°C and 37°C.

## Slide 8
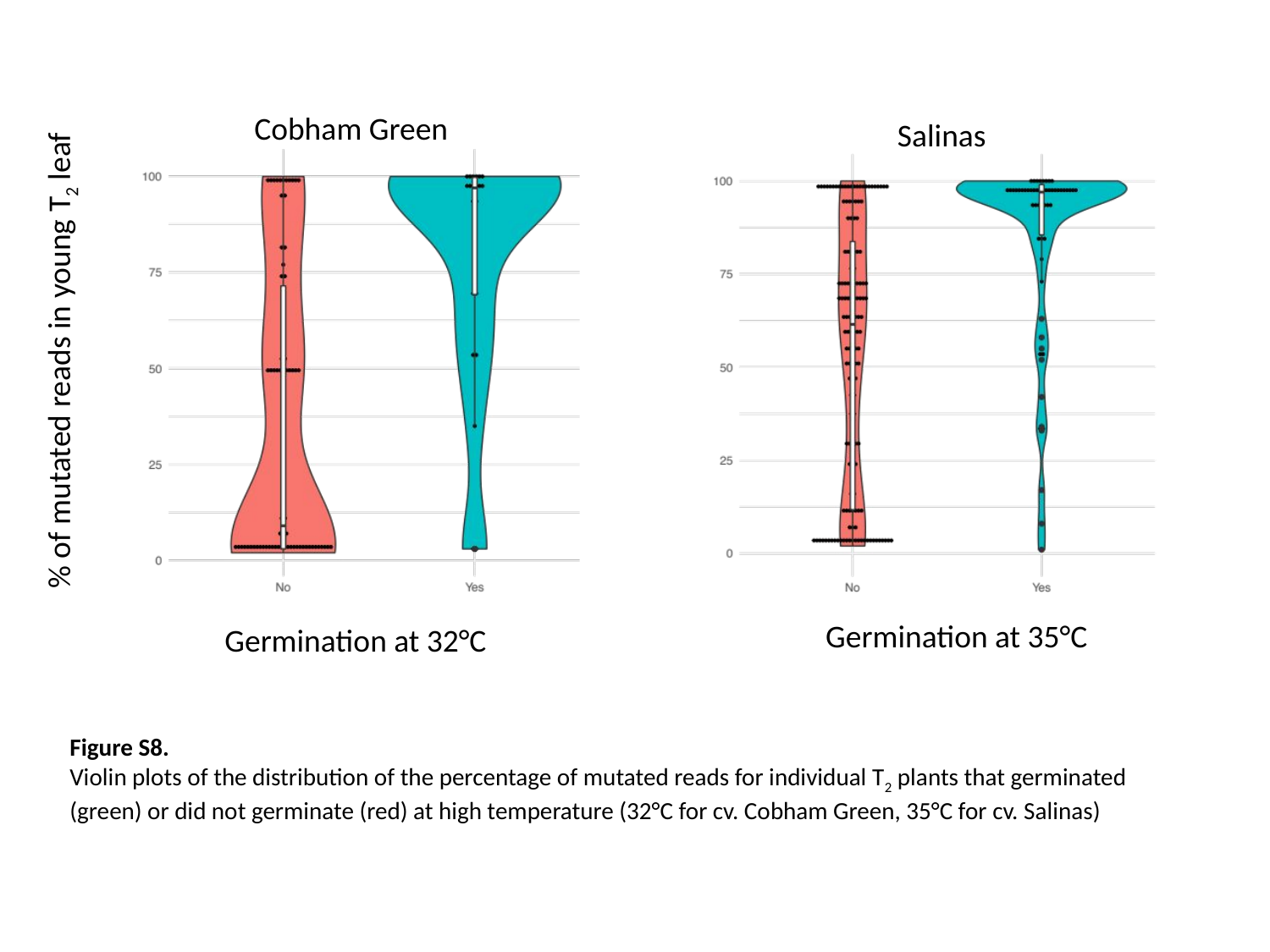

Cobham Green
Salinas
% of mutated reads in young T2 leaf
Germination at 35°C
Germination at 32°C
Figure S8.
Violin plots of the distribution of the percentage of mutated reads for individual T2 plants that germinated (green) or did not germinate (red) at high temperature (32°C for cv. Cobham Green, 35°C for cv. Salinas)
