# Supplementary material for: High-Resolution Analysis of the Efficiency, Heritability, and Editing Outcomes of CRISPR/Cas9-Induced Modifications of NCED4 in Lettuce (Lactuca sativa)
Source: G3 (Bethesda). 2018 Mar 29;8(5):1513–21. doi: 10.1534/g3.117.300396 (PMC5940144; doi:10.1534/g3.117.300396)
Supplement: Supplementary file 4 [file 1513FileS2.pptx]

## Slide 1
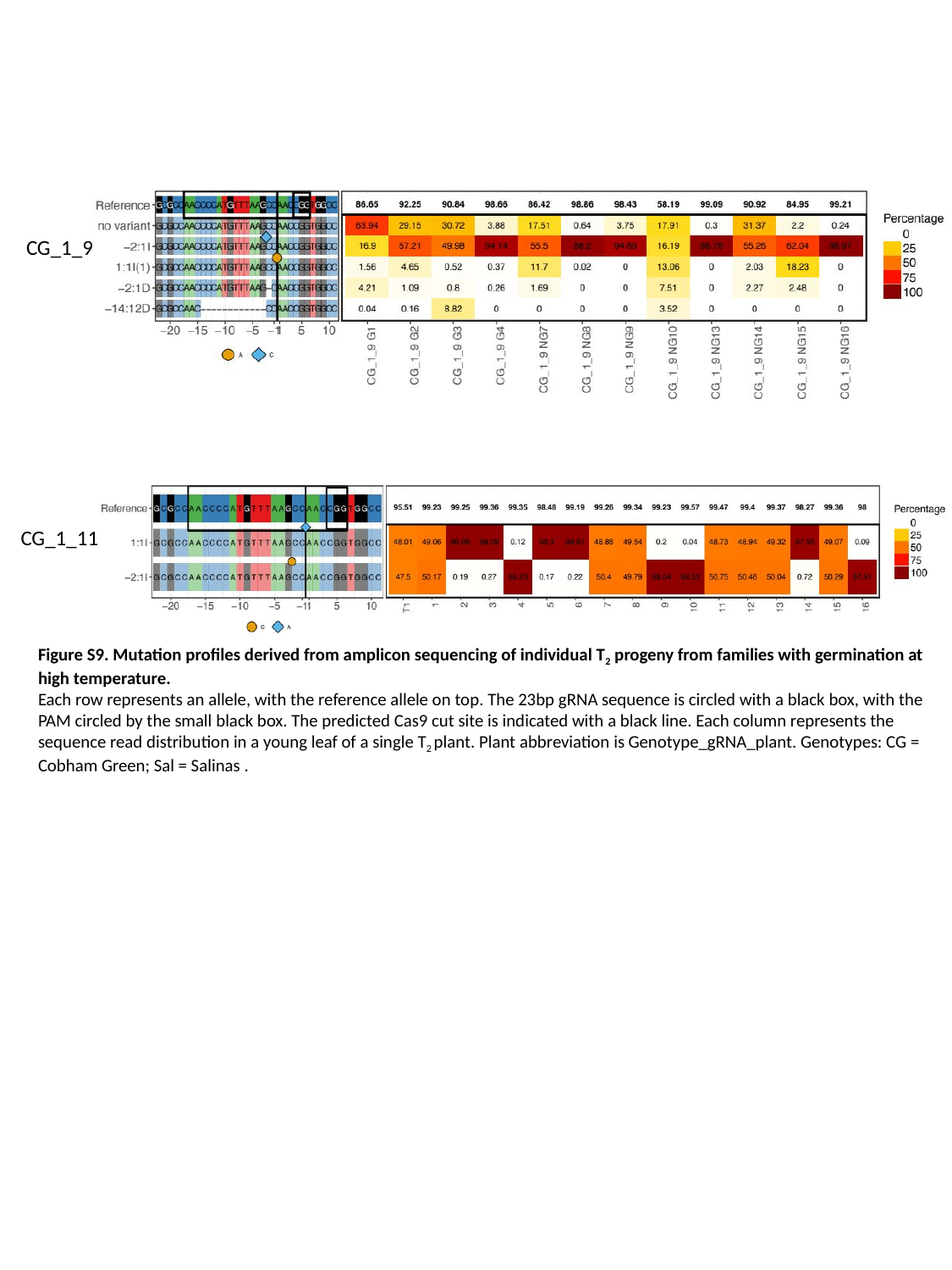

CG_1_9
CG_1_11
# Figure S9. Mutation profiles derived from amplicon sequencing of individual T2 progeny from families with germination at high temperature. Each row represents an allele, with the reference allele on top. The 23bp gRNA sequence is circled with a black box, with the PAM circled by the small black box. The predicted Cas9 cut site is indicated with a black line. Each column represents the sequence read distribution in a young leaf of a single T2 plant. Plant abbreviation is Genotype_gRNA_plant. Genotypes: CG = Cobham Green; Sal = Salinas .

## Slide 2
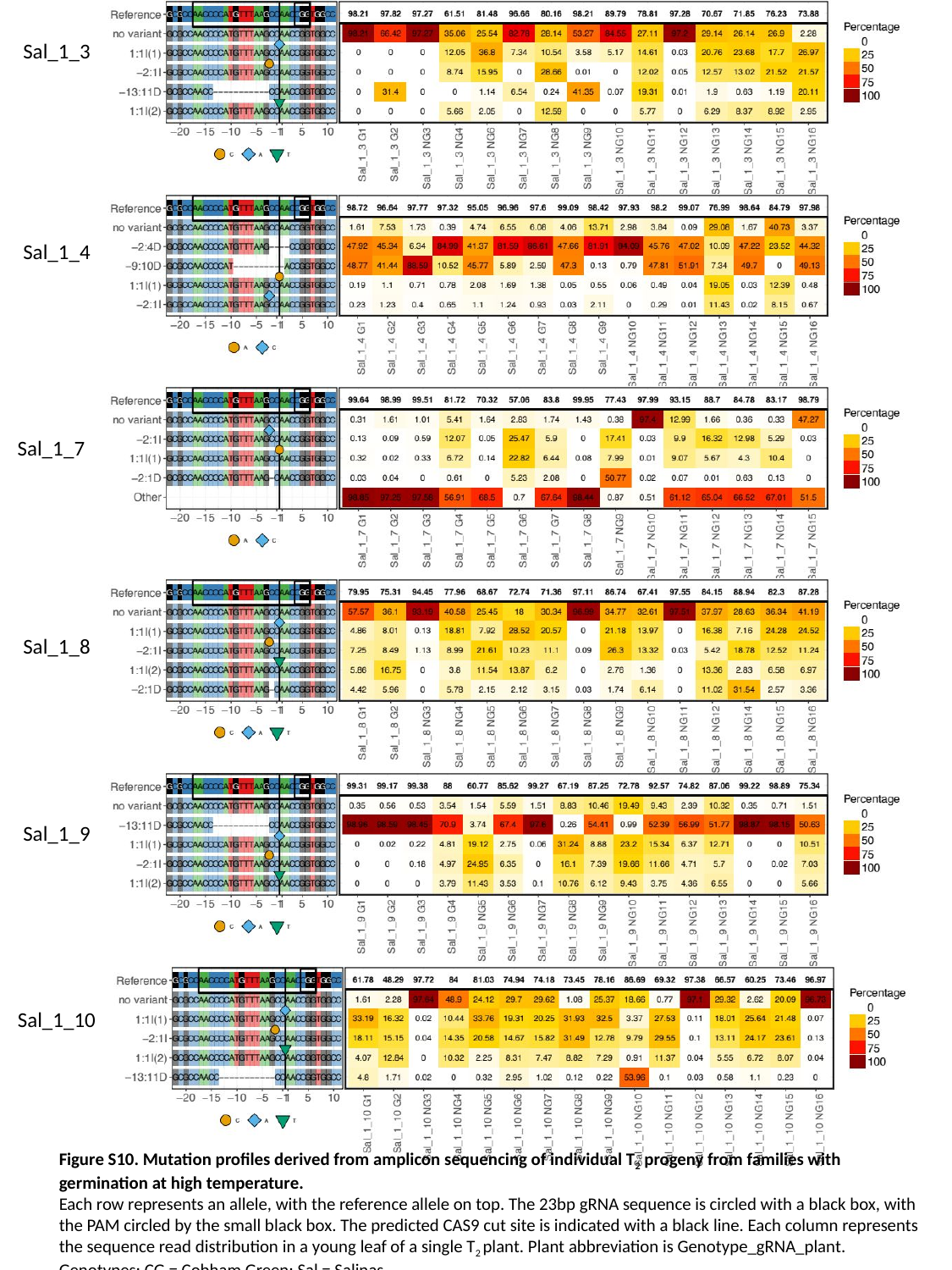

Sal_1_3
Sal_1_4
Sal_1_7
Sal_1_8
Sal_1_9
Sal_1_10
# Figure S10. Mutation profiles derived from amplicon sequencing of individual T2 progeny from families with germination at high temperature. Each row represents an allele, with the reference allele on top. The 23bp gRNA sequence is circled with a black box, with the PAM circled by the small black box. The predicted CAS9 cut site is indicated with a black line. Each column represents the sequence read distribution in a young leaf of a single T2 plant. Plant abbreviation is Genotype_gRNA_plant. Genotypes: CG = Cobham Green; Sal = Salinas .

## Slide 3
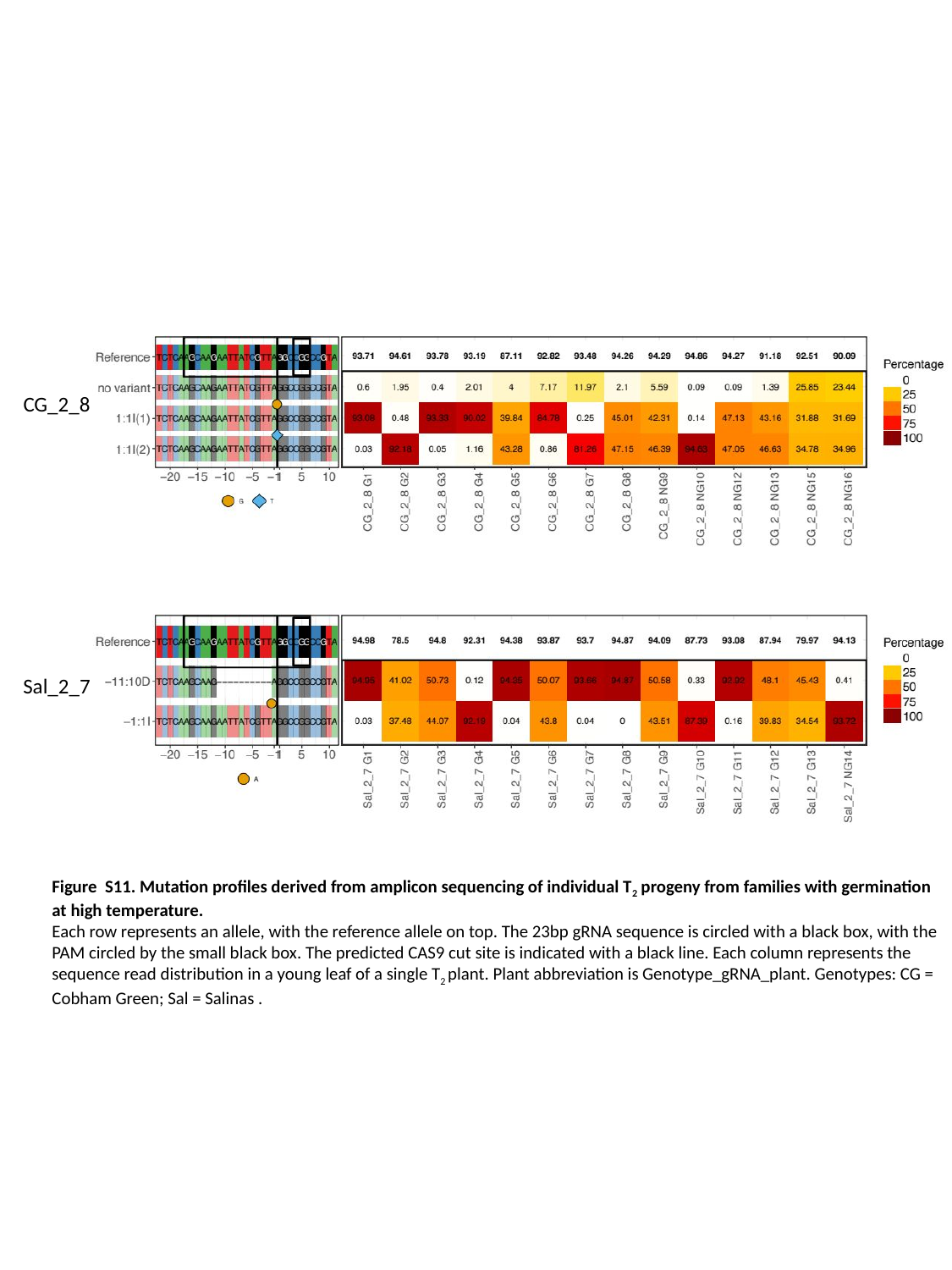

CG_2_8
Sal_2_7
Figure S11. Mutation profiles derived from amplicon sequencing of individual T2 progeny from families with germination at high temperature.
Each row represents an allele, with the reference allele on top. The 23bp gRNA sequence is circled with a black box, with the PAM circled by the small black box. The predicted CAS9 cut site is indicated with a black line. Each column represents the sequence read distribution in a young leaf of a single T2 plant. Plant abbreviation is Genotype_gRNA_plant. Genotypes: CG = Cobham Green; Sal = Salinas .

## Slide 4
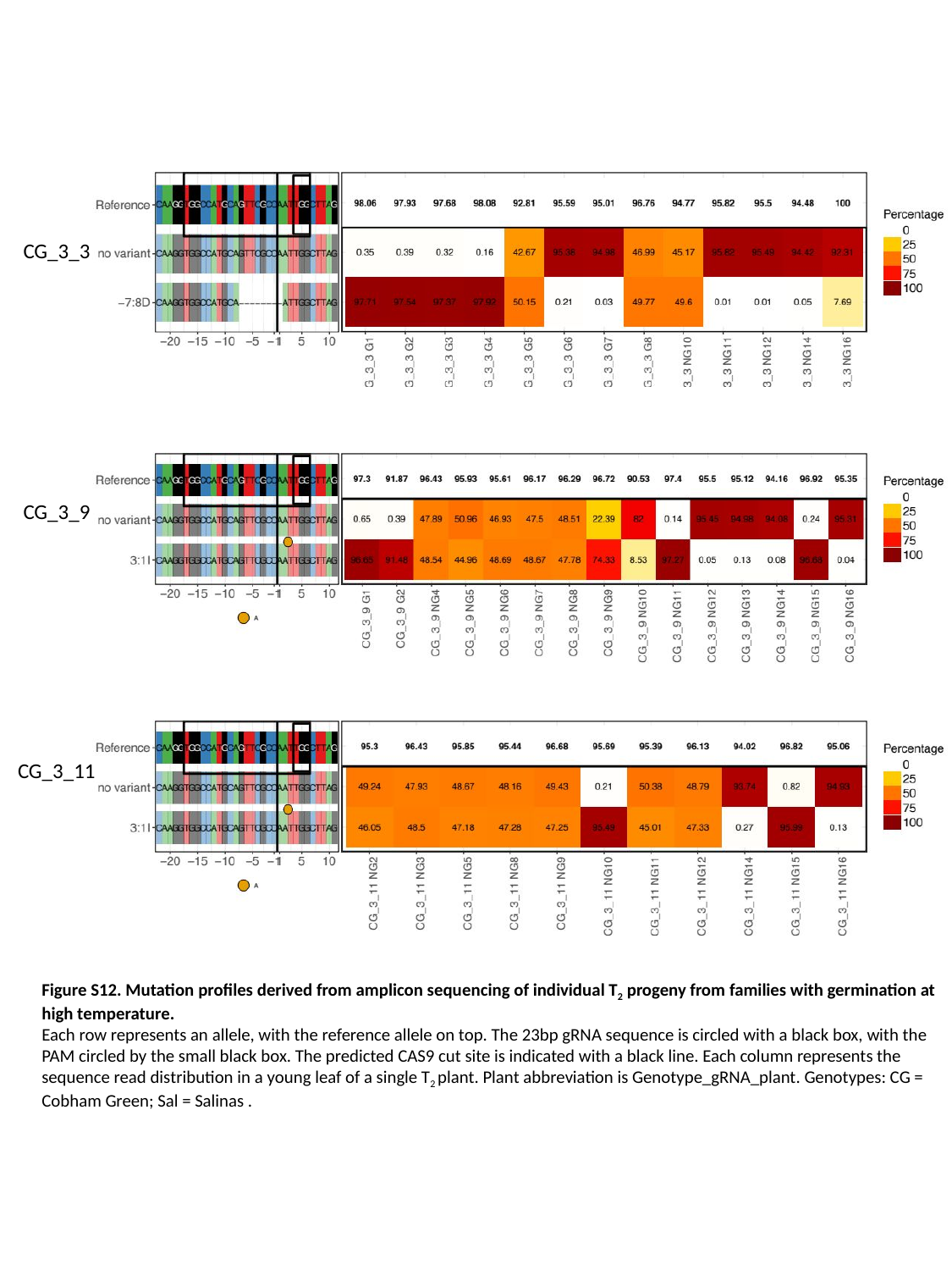

CG_3_3
CG_3_9
CG_3_11
# Figure S12. Mutation profiles derived from amplicon sequencing of individual T2 progeny from families with germination at high temperature. Each row represents an allele, with the reference allele on top. The 23bp gRNA sequence is circled with a black box, with the PAM circled by the small black box. The predicted CAS9 cut site is indicated with a black line. Each column represents the sequence read distribution in a young leaf of a single T2 plant. Plant abbreviation is Genotype_gRNA_plant. Genotypes: CG = Cobham Green; Sal = Salinas .

## Slide 5
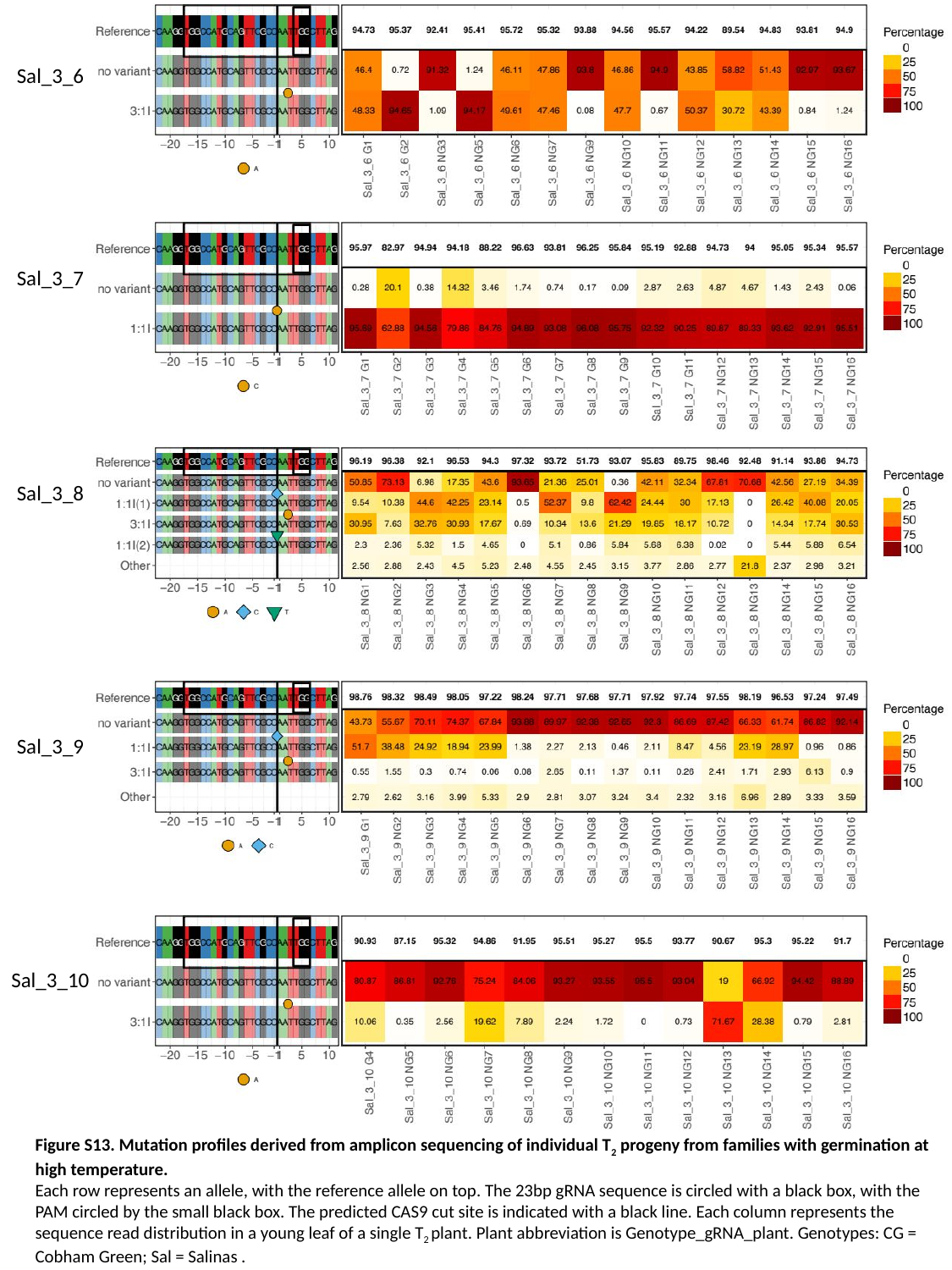

Sal_3_6
Sal_3_7
Sal_3_8
Sal_3_9
Sal_3_10
# Figure S13. Mutation profiles derived from amplicon sequencing of individual T2 progeny from families with germination at high temperature. Each row represents an allele, with the reference allele on top. The 23bp gRNA sequence is circled with a black box, with the PAM circled by the small black box. The predicted CAS9 cut site is indicated with a black line. Each column represents the sequence read distribution in a young leaf of a single T2 plant. Plant abbreviation is Genotype_gRNA_plant. Genotypes: CG = Cobham Green; Sal = Salinas .
